# Cholecystotomy, in One and Two Sittings

**Published:** 1895-03

**Authors:** J. McF. Gaston

**Affiliations:** Atlanta, Ga., Professor Principles and Practice of Surgery Southern Medical College, Atlanta, Ga.


### Southern Medical Record.

A MONTHLY JOURNAL OF MEDICINE AND SURGERY.

VOL. XXV. ATLANTA, GA., MARCH, 1895. No. 3.

### Original Articles.

### CHOLECYSTOTOMY IN ONE AND TWO SITTINGS.

BY J. McF. GASTON, M. D., ATLANTA, GA.,
Professor Principles and Practice of Surgery Southern Medical College, Atlanta, Ga.

A reprint from the Chicago *Medical Recorder* of January, 1895, has been received by the writer from Dr. Edward H. Lee, attending surgeon Cook County Hospital, Chicago, Ill., with a report of ten cases of cholecystostomy.

Six of these operations have been done by Dr. J. B. Murphy, three by Dr. J. H. Hoelscher and one by Dr. T. A. Davis, presumably in the above named hospital. Three have been performed in one sitting and seven in two sittings with various periods intervening between the first incision and the final opening of the gall-bladder. It is observed that this interval ranges from three to ten days. It is noted that the one which extended to ten days, was a case in which at the expiration of five days after packing with iodoform gauze without suture, some gauze had been placed between the two peritoneal surfaces, and that this had prevented the formation of adhesions at this point. The gauze was renewed, and after waiting another five days, the adhesions became complete, so that the secondary incision was made. The most striking feature of this report is that within the short periods of three, four and five days after packing with gauze the adhesions should be sufficient to warrant cutting into the sac. It is stated that anchor suture was employed in those running for three and five days, but that of four days interval had no stitches connecting the gall-bladder and points, and the exposed sac was only packed around with gauze. This strikes me as remarkable and indicates a plastic inflammation between the sewn surfaces which unites them in a less space of time than has generally been considered requisite for firm adhesion. If stitches were used to hold the surfaces closely in apposition, so that the plastic lymph or inflammatory exudate would not be disturbed by interference at this early stage, a secondary opening into the gall-bladder might be allowable within three or four days, but there being no urgency for this step, it would be preferable to wait at least eight days. It is in those cases, not presenting conditions which require immediate relief, that the operation in two sittings is employed; and the intention is to secure against any possibility of the escape of the contents of the sac into the peritoneal cavity. There is, therefore, no reasonable ground for this precipitation, and though it seems to have resulted favorably in these cases, it ought not to encourage less experienced operators than Dr. Murphy to repeat it. The operation in the case remaining only four days without suture of any kind, was interrupted at the outset by the effects of the anæsthetic, when the gall-bladder was brought forward into the abdominal incision. But while this prevented further operative procedure at that time, it does not appear that there was any cogent reason for making the secondary incision within four days, for the removal of gall stones from the gall-bladder and cystic duct. This instrumental interference at this stage of adhesion was calculated to break up the attachment between the sewn surfaces, and yet we are informed by the reporter that the patient made a rapid recovery, with only a small fistula.

In recapitulating these ten cases, it is stated that one case died three weeks after operation. This fatal termination was not due to the operation, but to the condition of the patient; to the advanced interstitial hepatitis and to the malignant growths that were present.

Another of the patients died three months after operation of another disease.

Of the remaining eight cases four have made a complete recovery; the other remaining four patients have still a fistula. But it is thought that these cases will result in a perfect recovery.

One of the cases which terminated fatally within three weeks, was subjected to the operation in two sittings; but there is nothing in the report to designate the other fatal result within three months, and, hence, it does not appear in evidence of the mode of procedure. But it is clear that none of the cases died from the effects of the operation, whether done in one or two sittings, and this corresponds to the statistics of cholecystotomy generally, as reported in latter years.

There may be a question in regard to the propriety of resorting to the operation in two sittings, in ordinary cases, and it is under such conditions Dr. Lee quotes me, as stating in the Reference Hand-book of Medical Science, "that the operation in two sittings has nothing to recommend it." He undertakes to combat my position, "that the most instructive digital examination of the gall-bladder and ducts is precluded by this procedure, and it affords no facilities for the removal of obstructions which may exist in the common and cystic duct." It is set forth by him that "the digital examination is not in any way precluded by this procedure. The time to examine the gall-bladder and the ducts is immediately after opening the abdominal cavity; the diagnosis of the pathological conditions is then to be made, and then the method of operation decided on."

Surely, it cannot be expected in the case of a large distended gall-bladder, that any satisfactory exploration of its contents, or the contents of the ducts, can be made without opening the sac, and this can be done with trocar and knife without escape of debris into the abdominal cavity. So soon as the fluid contents of the sac are evacuated, by the trocar, an incision into the gall-bladder now drawn outside of the parietal opening, admits of the introduction of the fingers or instruments, while a thorough digital examination outside of the sac is very much facilitated. Of course, it is expected that any gall-stones found within the sac shall be removed through the incision, and that if biliary concretions are found in the ducts, an attempt shall be made to force them from the common duct into the duodenum, and extract them in the cystic duct, through the gall-bladder. In the event these processes are not effective, then the only recourse is excision through the walls of the ducts, followed by suture of the opening or tamponage around, and drainage externally.

If cholecystotomy is performed in this mode, it offers great advantages, and, so far as the practice of cholecystostomy, in two sittings, by suturing the gall-bladder to the abdominal wall, before the sac is incised, which is defined by Dr. Lee, may be adopted, it must fail to secure these advantages.

I would not be understood as opposing, in all cases, chole cystotomy in two sittings, but, on the contrary, hold that some exceptional conditions call for this procedure, while, as a rule, it is still claimed that "the operation in two sittings has nothing to recommend it."

It may be that there is a typographical error on the fifth page of this reprint, in the statement that "if the cystic duct is free from obstructions, there is no indication for cholecystenterostomy." This is exactly the condition which warrants the operation when the common duct is impermeable; and yet Dr. Lee does not seem to recognize the obstruction of the ductus choledochus, and the free outlet for bile through the ductus cysticus, as the indication for cholecystenterostomy. This is not done, as supposed, for temporary relief, but as giving a permanent outlet of bile from the gall-bladder into the alimentary canal. The flow of bile through an external fistula brings many troubles in its train, while its entrance, by an artificial channel, into the duodenum, is beneficial in all respects. The practicability of this anastomosis was shown, ten years ago, by my experiments on dogs, and quite a considerable array of operations since on the human being, demonstrates that duodeno-cholecystotomy is recognized as a life-saving operation.

It is a source of much satisfaction to note the great benefits derived from the ingenious mode of anastomosis, devised by Dr. J. B. Murphy; and my contribution to the supplementary volume of the Reference Hand-book of Medical Science, contains a full account of the introduction of this spring button into gall-bladder surgery.

A modification of this button is alluded to by Dr. Lee for the purpose of drainage, and as I have personally witnessed the use of it by Dr. Murphy in a patient in the St. Joseph's Hospital at Chicago, it may be stated that it fulfilled the indications satisfactorily. By a tube communicating with the button which is fixed in the well of the gall-bladder, the collection is discharged beyond the external opening, thus effecting perfect drainage. As cholecystectomy is noticed in this report, as indicated where a large pedicled gall-bladder without adhesions is present, when there is gangrene of the gall-bladder, and in suitable cases of perforation of the cystic duct, it may be stated that my modication of the usual operation offers some advantages. This consists in cutting away the walls of the gall-bladder close to the hepatic attachment and leaving that portion of the sac which is adherent, without dissecting it off from the surface of the liver.

Recently I saw a case in consultation with Dr. Charles Whelan, at Birmingham, Ala., in which rupture of the sac had occurred some days previously; and I would have adopted this process had the condition of the patient permitted further operative procedure. But being almost moribund, the abdominal cavity was irrigated with hot water and the wound filled with iodoform gauze, as the only recourse. She died in a few hours, from the effects of general peritonitis, which had developed soon after the rupture of the gall-bladder occurred.

In referring to that procedure, which has generally been recognized by the term ideal cholecystotomy, Dr. Lee applies the new name, cholecystendysis, for this method. He considers it dangerous to remove a stone from the cystic duct by an incision in the sac; and after being assured of the freedom of the duct, to close the line of incision by suture and drop the gall-bladder into the abdomen. Yet this has been done successfully in Europe and in this country. A case was operated upon with a favorable result by Dr. V. O. Hardon, of Atlanta, and reported in my introductory address upon surgery of the gall-bladder and ducts, as chairman of the surgical section of the American Medical Association, at Detroit.

In one of the cases reported there was some confusion, in the diagnosis of the case as appendicitis until the patient was anæsthetized, when the gall-bladder could be felt. It is not likely that anything of this kind should occur again, but there have been a number of instances in which the kidney and gall-badder have been confounded in making a diagnosis and even so astute an observer as Dr. Goodell anchored the gall-bladder for a floating kidney.

